# L-2L ladder digital-to-analogue converter for dynamics generation of chemical concentrations

**DOI:** 10.1098/rsos.230085

**Published:** 2023-04-19

**Authors:** Tomohito Chatani, Suguru Shiraishi, Hiroki Miyazako, Hiroaki Onoe, Yutaka Hori

**Affiliations:** ^1^ Department of Applied Physics and Physico-informatics, Keio University, 3-14-1 Hiyoshi, Kohoku-ku, Yokohama, Kanagawa 223-8522, Japan; ^2^ Department of Mechanical Engineering, Keio University, 3-14-1 Hiyoshi, Kohoku-ku, Yokohama, Kanagawa 223-8522, Japan; ^3^ Graduate School of Information Science and Technology, The University of Tokyo, 7-3-1, Hongo, Bunkyo-ku, Tokyo 113-8656, Japan

**Keywords:** microfluidics, circuit analogy, fluid mechanics, reaction kinetics

## Abstract

Cellular response to dynamic chemical stimulation encodes rich information about the underlying reaction pathways and their kinetics. Microfluidic chemical stimulators play a key role in generating dynamic concentration waveforms by mixing several aqueous solutions. In this article, we propose a multi-layer microfluidic chemical stimulator capable of modulating chemical concentrations by a simple binary logic based on the electronic-hydraulic analogy of electronic R-2R ladder circuits. The proposed device, which we call L-2L ladder digital-to-analogue converter (DAC), allows us to systematically modulate 2^*n*^ levels of concentrations from single sources of solution and solvent by a single operation of 2*n* membrane valves, which contrasts with existing devices that require complex channel geometry with multiple input sources and valve operations. We fabricated the L-2L ladder DAC with *n* = 3 bit resolution and verified the concept by comparing the generated waveforms with computational simulations. The response time of the proposed DAC was within the order of seconds because of its simple operation logic of membrane valves. Furthermore, detailed analysis of the waveforms revealed that the transient concentration can be systematically predicted by a simple addition of the transient waveforms of 2*n* = 6 base patterns, enabling facile optimization of the channel geometry to fine-tune the output waveforms.

## 1. Introduction

The complex interplay of biomolecular reactions enables the adaptive response of biological systems to exogenous stimuli in surrounding environments. Microfluidic devices are suitable platforms for systematically studying the kinetics of the underlying reaction pathways by dynamically modulating the chemical stimuli and measuring the dynamic response of the biological process within a controlled compartment [1,2]. Previous studies performed dynamic stimulation to various types of cells to probe signalling mechanisms of cells such as calcium signalling [3–5], immune signalling [6–8] and neural activities [9]. Other studies demonstrated the temporal control of gene expression by modulating the corresponding inducer concentrations over time [10–16]. These studies suggested that microfluidic chemical stimulators play a crucial role not only in the system-level understanding of biological process but also in the development of bioengineering applications such as protein synthesis, drug screening and stem cell engineering.

To date, many types of microfluidic chemical stimulators were developed to meet different needs of resolution, precision and response time. In the case of binary temporal stimulations, a popular approach is to use laminar flow and regulate its boundary position to apply the chemical stimulus to the target specimens [17–19]. For multi-level stimulations, branch channels are used to generate the gradient of concentrations in parallel microfluidic channels [4,20–22]. These approaches are suited for generating stimulus patterns at a predefined concentration over time. However, the flexibility of the concentration waveforms is limited due to the lack of dynamic regulation mechanisms of channel flow rates. To overcome this limitation, recent studies explored dynamic mixing mechanisms of several aqueous solutions to make desired concentrations at high resolution. These techniques are called ‘function generator’ or ‘digital-to-analogue converter’ of chemical concentrations named after its circuit analogy in electrical engineering. Some existing microfluidic digital-to-analogue converters (DACs) used gravity pumps [7,13,23], and pressure regulators [3] that modulate the flow rates or inlet pressures of multiple solutions to be mixed. Others adopted on-chip membrane valves [24] that can control the flow lines based on a series of well-defined valve operations to achieve higher precision of dynamic concentration control [25,26]. The on-chip valve enabled many concepts of electric circuits such as pulse width modulation (PWM) and serial DAC to be translated into microfluidic channels [6,27–30]. Among them, the DACs proposed by Chen *et al.* [31] and Piehler *et al.* [6] had an advantage that a target concentration could be generated only by a single operation of on-chip valves. Thus, their devices achieved not only high precision but also fast response, which is particularly important for applications that need high-frequency stimulation. However, a caveat is that their devices had complex channel geometries with many junctions or control valves to generate dilution series of a single solution [31] or to mix multiple solutions from many inlets [6]. Moreover, the control logic of the valves in the study by Piehler *et al.* [6] required empirical tuning by computational simulations, which would give only suboptimal solutions with no interpretations. Thus, redesign and re-optimization of these DACs for different resolutions would be hindered.

In this article, we propose a simple microfluidic DAC capable of modulating dynamic waveforms of chemical concentrations using an intuitive binary logic of on-chip membrane valves. The mechanism of the waveform modulation is based on the analogy of an electronic digital-to-analogue conversion circuit called the R-2R ladder network. Specifically, the proposed device uses 2*n* membrane valves to regulate the chemical flow from inlets and convert the concentration of a single solution into a desired level. In theory, the proposed device, which we call L-2L ladder DAC, can generate 2^*n*^ levels of concentrations using only 2*n* membrane valves and single sources of solution and solvent, attaining higher resolution with fewer input sources than the existing DAC devices. Moreover, the response time is within the order of seconds, since the concentration levels can be switched by a single operation of membrane valves. To verify these concepts, we fabricated the L-2L ladder DAC with *n* = 3 bit resolution. Experimentally, we performed dynamic generation of 2^3^ = 8 levels of concentrations from single sources of solution and solvent and confirmed the digital-to-analogue conversion by comparing the modulated concentrations with the computational simulations. The detailed analysis of the waveforms further revealed that the transient response of the DAC can be systematically predicted by simple addition of 2*n* = 6 base transient patterns. This feature allows for facile modification of channel geometry to fine-tune the output of the device, which is particularly important for scaling up the device for higher resolution.

## 2. Device concept

**Figure 1.**
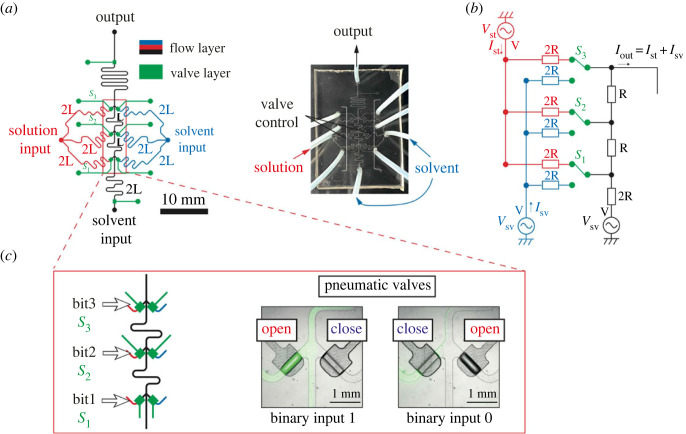
(*a*) Top view of L-2L ladder DAC with membrane valves. The flow line is shown in black, red and blue, and the control line is in green. Pneumatic pressure was applied to each inlet port shown by coloured circles and was controlled by solenoid valves as needed. The mixed solution was measured near the outlet port (output) of the device. (*b*) R-2R ladder resistor network with 3-bit resolution for DAC. The circuit has two voltage sources *V*_st_ and *V*_sv_ with the same voltage corresponding to the constantly pressurized solution and solvent inputs in the hydraulic system, respectively. The current flow from each voltage source denoted by *I*_st_ and *I*_sv_ is determined by the configuration of the 3-bit switches (*S*_1_, *S*_2_, *S*_3_). (*c*) Enlarged view of the membrane valves. The input signal is configured by opening one of the two symmetrical valves.

We designed the microfluidic DAC in figure 1*a* based on the electronic–hydraulic analogy (table 1) [32] of an electronic DAC circuit called R-2R resistor ladder network in figure 1*b*. The designed microfluidic device was equipped with membrane valves controlled by pneumatic pressure. The membrane valves serve as an analogue of the switches in the electric circuit in figure 1*b* and allow for the selection of flow patterns from the two inlets, whose pressures corresponding to the voltage sources are set constant. The flow resistance *R*_H_ in table 1 is proportional to the length of the flow channel *L* when the difference of the viscosity Δ*η* between the solution and the solvent is negligibly small, and the flow channel has a uniform cross-sectional shape. Based on this observation, the flow resistance corresponding to the electric resistors *R* and 2*R* in figure 1*b* was carefully crafted by adjusting the length of the flow channel to *L* and 2*L*, respectively. Thus, the microfluidic device is named L-2L ladder DAC.

**Table 1.** Electronic–hydraulic analogy. The symbols *η*, *S*, *C* and *L* represent the viscosity of fluid, the cross-sectional area of channel, the circumference of channel and the length of channel, respectively.

electric circuit	hydraulic circuit
voltage	pressure
*V* (V)	Δ*P* (N m^−2^)
current	volume flow rate
*I* (A)	*Q* = (*π*Δ*P*/8*ηL*)(2*S*/*C*)^4^ (m^2^ s^−1^)
resistance	flow path resistance
*R* (Ω)	*R*_H_ = (8*ηL*/*π*)(*C*/2*S*)^4^ (Pa s m^−1^)

The mechanistic basis of the digital-to-analogue conversion can be verified by a simple circuit analysis of the R-2R resistor ladder network. In figure 1*b*, the three switches (*S*_1_, *S*_2_, *S*_3_) regulate the ratio of the current flow from the two constant voltage sources *V*_st_ and *V*_sv_ in a linear fashion depending on the switch configurations, which, in hydraulic systems, corresponds to mixing two constantly pressurized solutions at linearly divided flow rates. Specifically, the output current *I*_out_ is calculated as follows:

2.1 $$\begin{array}{rl} & I_{\text{out}}=I_{\text{st}}+I_{\text{sv}}\\ \mathrm{and}\; & I_{\text{st}}=\sum_{k=1}^{3}2^{k-1}S_{k}I,\;\;I_{\text{sv}}=I+\sum_{k=1}^{3}2^{k-1}(1-S_{k})I, \end{array}\}$$

where *I*_st_ and *I*_sv_ represent current from *V*_st_ and *V*_sv_ to the output, respectively, *I* is a constant defined by *I* = (1/2)^3^ × (*V*/*R*) and *S*_*k*_ (*k* = 1, 2, 3) are variables for the switches defined as follows:

$$S_{k}=\left\{ \begin{array}{cc} 1\; & \text{( Switch }k\text{ is connected to }V_{\mathrm{st}})\;\\ 0\; & (\text{Switch }k\text{ is connected to }V_{\mathrm{sv}}).\; \end{array}\right.$$

Equation (2.1) implies that the current flowing from the two voltage sources is linearly divided into eight levels corresponding to the bit patterns (*S*_1_, *S*_2_, *S*_3_) of the switches. In other words, the current flow from two input sources can be controlled by the simple binary logic. Recalling the electronic-hydraulic correspondence of electric current and flow rate, this means that the concentration at the outlet of the microfluidic device can be controlled at eight levels, i.e. 3-bit resolution, by the open/close combinations of the three membrane valves as illustrated in figure 1*c*. In particular, the channel topology of the L-2L ladder DAC can be easily generalized for the generation of 2^*n*^-level concentration patterns using 2*n* switches.

## 3. Results and discussion

**Figure 2.**
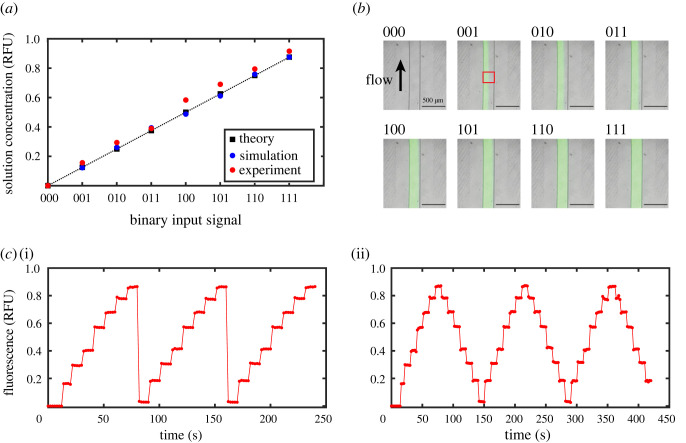
(*a*) Solution concentration for each configuration of membrane valves. Concentration was regulated with 3-bit resolution. Relative fluorescence unit (RFU) is normalized by the fluorescence intensity of the stock fluorescein solution applied to the solution inlet. (*b*) Fluorescence images near the outlet showing regulated solution–solvent ratios. Mean fluorescence intensity was quantified using the area shown by the red square. (*c*) Dynamic control of fluorescein concentrations by L-2L ladder DAC: (i) sawtooth wave and (ii) triangular wave.

To verify the generation of the concentration patterns, we performed computational fluid simulations of the designed microfluidic device. Specifically, the concentration at the outlet port was computed for the eight different configurations of the valve patterns (see electronic supplementary material). The computed concentration agreed with the 3-bit binary patterns of the valve configurations and showed good linearity as shown in figure 2*a*. This suggests that highly accurate digital-to-analogue conversion of chemical concentration is possible with the resolution of 3 bits or higher using the L-2L ladder DAC.

Motivated by this observation, we fabricated the microfluidic device in figure 1*a* using polydimethylsiloxane (PDMS) and verified the concentration patterns at the outlet of the device. For the verification purpose, a fluorescein solution and double distilled water (ddH_2_O) were prepared as the solution and the solvent input applied to the proposed device, respectively. Each input solution was placed in a reservoir tube that was pressurized at 100 kPa for constant pressure pumping. Then, the membrane valves were regulated by pneumatic pressure to generate the eight configurations of the valve patterns using a computer-controlled custom electric circuit. After the flow was stabilized, the fluid channel near the outlet port was observed with an inverted fluorescence microscope. As shown in figure 2*b*, we observed laminar flow with different ratios of the solution and the solvent depending on the configuration of the membrane valves. It is noted that this laminar flow would be mixed in a downstream channel to make a homogeneous aqueous solution using passive or active mixing techniques [33]. Thus, we here analysed the mean fluorescence intensity of the flow channel indicated by the red rectangular in figure 2*b* to quantify the output concentration. As illustrated in figure 2*a*, the overall trend of the output matched with the valve patterns in almost a linear fashion, suggesting that the L-2L ladder DAC could perform digital-to-analogue conversion of chemical concentrations based on the binary patterns of the membrane valves. It should be noted that the relatively large discrepancy between theory and experiment for 100, 101, 110 and 111 is probably due to a fabrication error that originates from the inaccuracy of the mould milling process and/or multi-layer soft lithography, which involves more steps than a single-layer standard soft lithography (see §5). In general, the achievable resolution of the output, i.e. the number of bits achievable, is limited by the accuracy of the output that is determined mainly by the fabrication accuracy of the PDMS replica.

Next, we tested the dynamic regulation of the solution concentration by switching the valve configurations within a short period of time. Specifically, we switched the configuration patterns every 10 s and generated the sawtooth wave and the triangular wave. Figure 2*c* shows the output concentration for three cycles of the periodic waves. These results imply that the profile of the periodic waves is highly reproducible and the concentration quickly reaches the target value within a few seconds after switching the values, suggesting that the L-2L ladder DAC can be used as a dynamic stimulus generator for biological applications.

**Figure 3.**
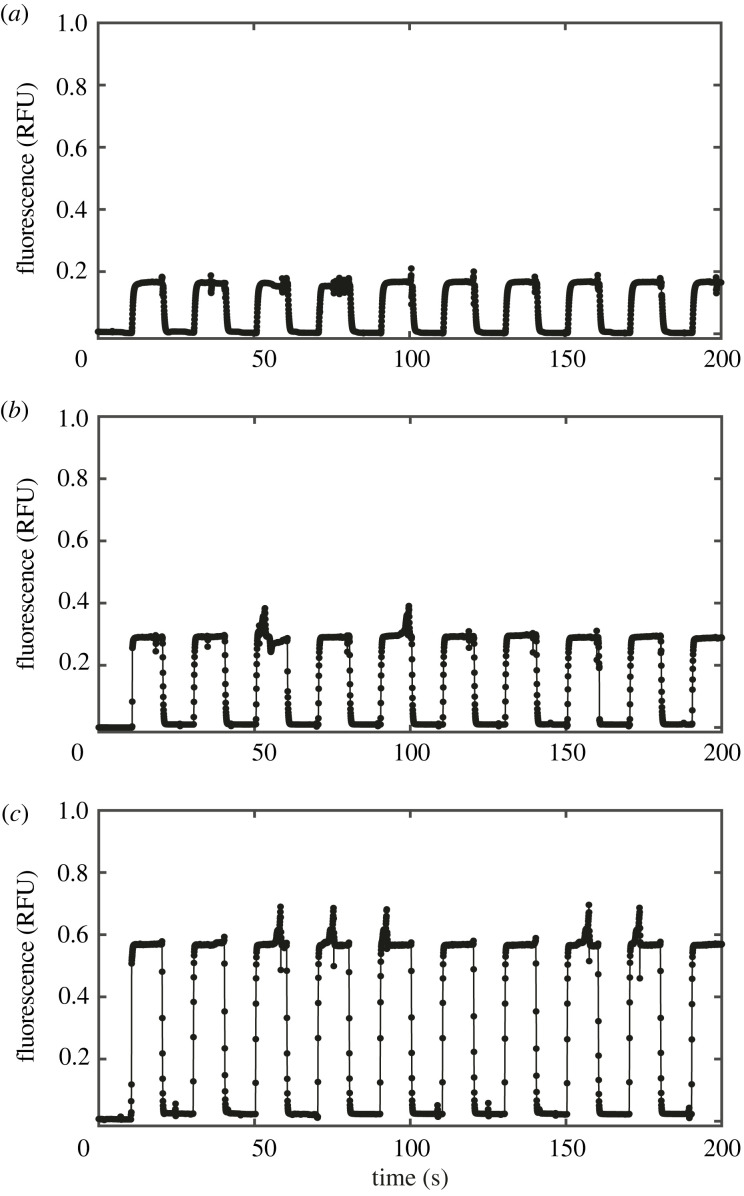
Multiple cycles of rise and fall steps for (*a*) 000 ↔ 001, (*b*) 000 ↔ 010 and (*c*) 000 ↔ 100.

To quantitatively characterize the transient dynamics of the output concentration, we next measured the rise and the fall steps of the outlet concentration when a single bit was switched to/from 1 from/to 0, i.e. 000 ↔ 001, 000 ↔ 010 and 000 ↔ 100. Specifically, the valves were switched every 10 s for more than 10 cycles, and the fluorescence at the outlet was measured at the sampling period of 50 ms. The result in figure 3 showed that the output fluorescence was highly reproducible for more than 10 cycles. The slightly biased baseline fluorescence for the input 000 after the second cycle was presumably due to a residual compound since the output tended to decrease in time during the input, which could be improved by surface treatment. On the other hand, the response time of the output was variable depending on the position of the valve to be opened/closed.

**Figure 4.**
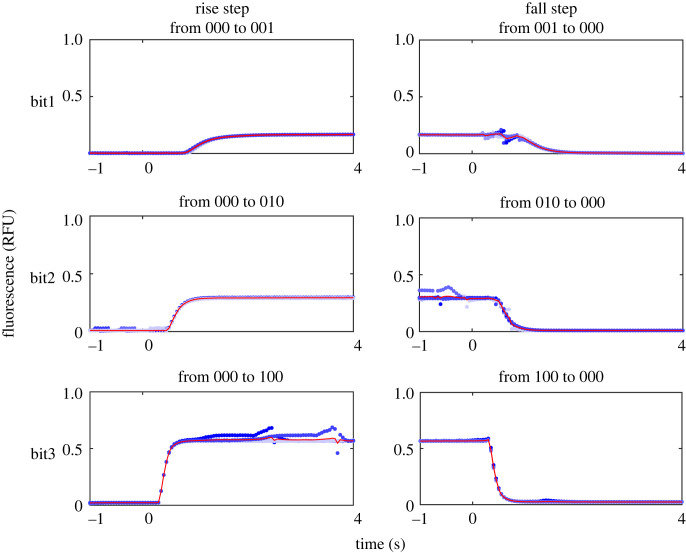
Transient behaviour of base switching patterns. Original time-series data (five repeats) are shown in blue, and their averaged response is shown in red.

**Figure 5.**
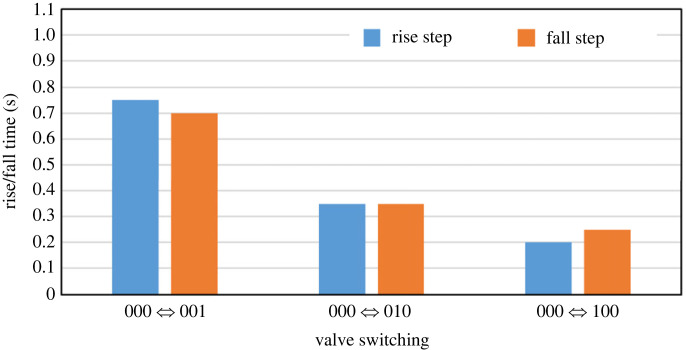
Rise and fall time for base switching patterns.

The variability of the response time could be observed more clearly in figure 4 by averaging multiple rise and fall steps of figure 3. In particular, we observed the trend that the rise/fall time, i.e. time to change from/to 10% to/from 90% of the final/initial value, was shorter for the higher-order bits as summarized in figure 5. These trends were probably caused by the difference of the channel lengths from the membrane valves to the outlet. Specifically, the membrane valve for a higher-order bit was located closer to the outlet (figure 1*a*), and moreover, the rate of inflow at the valve was larger for higher-order bits, as can also be verified from the analogy circuit in figure 1*b* by calculating the incoming current from each switch. Consequently, the input solutions from high-order bits arrived earlier than those from low-order bits, resulting in the shorter rise/fall time.

**Figure 6.**
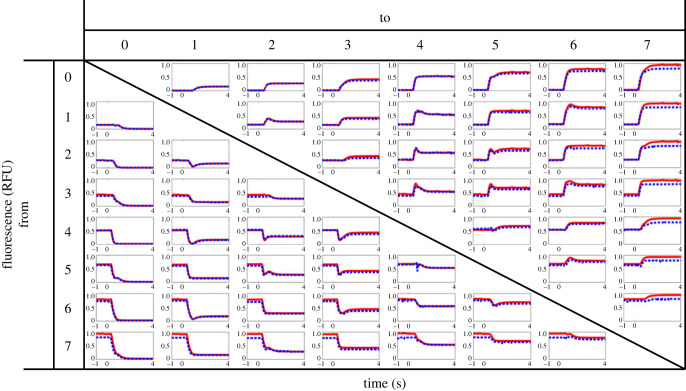
Transient responses for all of the 28 switching configurations. Computational predictions are shown in red, and experimental results are shown in blue.

To comprehensively characterize the transient dynamics of the output concentrations, we further measured the time-series data of the output fluorescence for all the other possible combinations of the valve switching patterns as shown by the red lines in figure 6. The output fluorescence converged to the final value within 2 s for all of the 28 switching patterns. This implies that the L-2L ladder DAC is capable of generating complex dynamic waveforms that vary in the order of seconds. Another observation from figure 6 was that the output concentration could overshoot or undershoot depending on the switching patterns of the valves.

We found that these transient behaviours could be rationally characterized by the output of the base switching patterns in figure 4. Specifically, all possible switching patterns in figure 6 can be expressed as the addition of the *base* switching patterns in figure 4 (000 → 001, 000 → 010, 000 → 100, 001 → 000, 010 → 000 and 100 → 000). Thus, we can computationally add the experimental time-series data in figure 4 to predict the output fluorescence in figure 6. For example, 2 → 4 (010 → 100) can be predicted by adding the rise step of bit 3 and the fall step of bit 2. The computationally predicted fluorescence is shown by the red solid curves in figure 6. The predicted time-series data in each panel (red) matched with the experimental data (blue), including the instant overshoot/undershoot with high accuracy. These results implied that the transient dynamics of the output such as dead time, rise/fall time and overshoot/undershoot could be systematically characterized by the small number of the base switching patterns in figure 4. Moreover, the results support our hypothesis that the rise/fall time is determined by the channel length from a membrane valve to the outlet, since the response time for flipping a single bit is almost the same regardless of the configuration of the other bits. For example, in the panel 2 → 3, the blue line shows an experimental result for 010 → 011, while the red line in the same panel shows a response curve for the base switching pattern 000 → 001, i.e. with a biased initial value. Since these lines matched with each other, the difference in response time was dependent only on the flipped bit. These observations allow us to further improve the shape of the response curve by optimizing the timing of the valve operation if necessary.

These mechanistic characterizations of the output concentration enabled systematic modulation of dynamical chemical waveforms using the simple channel geometry (figure 1) and the intuitive binary control logic (equation (2.1)). In particular, the L-2L ladder DAC has two remarkable features that (i) the device can generate 2^*n*^ levels of solution concentrations only from single sources of solution and solvent using 2*n* switches, and (ii) the waveforms can be controlled by an intuitive binary logic that is theoretically supported by the electronic-hydraulic analogy. These features contrast with existing devices that have many junctions for mixing multiple input solutions [31] and require tuning of the valve control logic with advanced computational tools [6]. Moreover, the systematic predictability of the transient dynamic concentration shown in figure 6 allows us to separately optimize the single channels for fine-tuning the transient waveforms. Thus, the L-2L ladder DAC is amenable to scale-up for a higher resolution.

In practice, the resolution of the output, i.e. the number of bit, should be determined based on the accuracy of the output (figure 2*a*) that depends mainly on the fabrication accuracy of the mould and the PDMS replica. The transient fluctuations such as those observed in figures 3*c* and 4 were possibly due to residual compounds that stagnated at the junctions of the channels near the valves and could be reduced by making the confluence of the flow paths at a sharper angle. In experiments, one should consider the difference in viscosity between solution and solvent, since our theoretical analysis is founded on the assumption that the difference in viscosity is negligibly small. In addition, the inlet pressure should also be set high enough to minimize the effect of unmodelled factors such as the inlet resistance.

## 4. Conclusion

We have proposed a simple microfluidic device called L-2L ladder DAC for generating dynamic waveforms of chemical concentrations from single sources of solution and solvent. The design of the proposed device is based on the analogy of an electronic DAC, and thereby the output concentration is theoretically characterized by the combinations of the binary mixing patterns of solution and solvent. The L-2L ladder DAC is highly flexible in terms of the resolution of the output concentration since the resolution can be increased exponentially against the number of membrane control valves. Moreover, the response time to reach the set point is within the order of seconds since the output concentration is configured by a single operation of membrane valves. These features allow the device to be used in diverse application fields in biology and chemistry that requires high-frequency stimulus for interrogating fast kinetics. Furthermore, the in-depth study of the waveforms has revealed that the transient concentration of the L-2L ladder DAC can be systematically predicted by the sum of the waveforms for the base switching patterns. These systematic characterizations facilitate the fabrication of the proposed device with desired precision and resolution.

## 5. Material and methods

### 5.1. Computational fluid simulation

The computational fluid simulation in figure 2 was performed using the COMSOL Multiphysics software^®^ with the microfluidics module. Two-dimensional geometry of the flow channel was rendered using AutoCAD and was imported into the software for the simulation. The width and the height of the channel were set to 200 and 20 μm for the entire device, respectively. The flow rate of the solution was calculated based on the Navier–Stokes equation in the laminar flow interface as the convection term in the diffusion equation in the diluted species transport interface. Specifically, the laminar flow interface and the transport of diluted species interface were used to solve the equation. Laminar flow was assumed, and the gravity term was neglected. The diffusion solvent was set as water, and the diffusion coefficient was set to 1.0 × 10^−9^ m^2^ s^−1^.

### 5.2. Device fabrication

The microfluidic device was fabricated by multi-layer soft lithography using polydimethylsiloxane (PDMS; SILPOT 184, Dow Corning Toray). The device consisted of two layers: the upper layer for the control of the pneumatic valves and the lower layer for the flow of solutions. Acrylic moulds were made for each layer using a milling machine (NMV-1500DCG, DMG MORI). The width and the height of the flow channels and the control channels were designed to be 200 and 20 μm, respectively. The width and the height of the membrane valves were designed to be 800 and 80 μm, respectively. The PDMS base and curing agent were mixed at a ratio of 5 : 1 for the upper layer and 20 : 1 for the lower layer, and the mixtures were degassed using a vacuum desiccator. The mixture for the lower layer was then spin-coated on the lower-layer mould at 300 r.p.m. for 30 s and then 1500 r.p.m. for 30 s. The mixture for the upper layer was poured on the upper-layer mould. Both moulds were soft baked at $80^{\circ }C$ for 30 min. After the soft bake, the upper-layer PDMS was immediately peeled off from the mould, and the inlet holes were punched with a biopsy punch with 1.5 mm hole (Kai industries). The punched PDMS replica was then placed on the lower-layer mould for curing at $85^{\circ }C$ for 3 h. The cured PDMS was then peeled from the mould, and the inlet holes were punched using the biopsy punch with 1.5 mm diameter hole. Finally, the PDMS replica was bonded with an ultrasonically cleaned glass slide using plasma for 10 s using a plasma irradiation device (PC-400T, Strex). The device was further heat cured overnight to improve the bond strength.

### 5.3. Experimental details

Fluorescein was purchased from FUJIFILM Wako Pure Chemical Corporation and was dissolved in ddH_2_O to make the solution at 2.0 × 10^−3^ g l^−1^. The fluorescein solution and ddH_2_O were used as the solution and solvent input for all the experiments, respectively. In all experiments, each solution was placed in a centrifuge tube with a reservoir tube cap (HE0200, System Biotics). A pneumatic regulator connected to an air compressor was set to 100 kPa to pump the solution from each centrifuge tube at a constant pressure. The valve control channels were connected to another pneumatic regulator set to 250 kPa. The on-off switching of the pneumatic pressure was then controlled by solenoid valves using a custom circuit board connected to a computer. The flow channel was washed with the solvent (ddH_2_O) by configuring the input as 000 before each experiment. Then, fluorescence near the outlet of the device was measured. In figure 2*a*, the membrane valves were kept for 60 s for each input pattern, and the average fluorescence intensity was calculated using data from 10 to 60 s. In figure 4, the mean response curves shown in red were plotted by averaging the five responses shown in blue. In figure 5, rise/fall time was determined as the time between 0.9*x*_max_ and 0.1*x*_min_, where *x*_max_ and *x*_min_ are the mean response value for 3–4 s and −5–0 s, respectively.

### 5.4. Data acquisition

Solution fluorescence in the microfluidic device was imaged using Nikon Ti–S microscope with an sCMOS camera (pco.panda, Excelitas PCO) at 20 frames per second with exposure time of 45 ms for each frame. The image acquisition was done by synchronizing a multi-functional DAQ board (USB6212, NI) with the pneumatic control system. The raw data were analysed using ImageJ [34] to measure a 200 μm square area in the channel near the output port and to calculate the average intensity value. In all figures, the fluorescence intensity on the vertical axis was subtracted by the background intensity measured by flowing ddH_2_O and was normalized by the stock fluorescence intensity at the concentration of 2.0 × 10^−3^ g l^−1^.

## Data Availability

The data and codes are available at https://github.com/hori-group/L-2L-ladder-DAC. Simulation results are provided in electronic supplementary material [35].
